# From Whole-Brain Radiotherapy to Immunotherapy: A Multidisciplinary Approach for Patients with Brain Metastases from NSCLC

**DOI:** 10.1155/2019/3267409

**Published:** 2019-02-03

**Authors:** Maria Protopapa, Vassilis Kouloulias, Styliani Nikoloudi, Christos Papadimitriou, Giannis Gogalis, Anna Zygogianni

**Affiliations:** ^1^National and Kapodistrian University of Athens, Medical School, Radiation Oncology Unit, 1st Department of Radiology, Aretaieion University Hospital, Greece; ^2^National and Kapodistrian University of Athens, Medical School, Radiation Oncology Unit, 2nd Department of Radiology, Attikon University General Hospital, Greece; ^3^National and Kapodistrian University of Athens, Medical School, Medical Oncology Unit, 2nd Surgery Clinic, Aretaieion University Hospital of Athens, Greece

## Abstract

Non-small cell lung cancer patients with brain metastases have a multitude of treatment options, but there is currently no international and multidisciplinary consensus concerning their optimal treatment. Local therapies have the principal role, especially in symptomatic patients. Advances in surgery and radiation therapy manage considerable local control. Systemic treatments have shown effect in clinical trials and in real life clinical settings; yet, at present, this is restricted to patients with asymptomatic or stable intracranial lesions. Targeted agents can have a benefit only in patients with EGFR mutations or ALK rearrangement. Immunotherapy has shown impressive results in patients with PD-L1 expression in tumor cells. Its effects can be further enhanced by a synergy with radiotherapy, possibly by increasing the percentage of responders. The present review summarizes the need for more effective systemic treatments, so that the increased intracranial control achieved by local treatments can be translated in an increase in overall survival.

## 1. Introduction

Lung cancer remains the leading cause of cancer death, with 53% of new lung cancer diagnoses being metastatic, when the 5-year relative survival rate is only 5% [1–3]. The central nervous system (CNS) is together with the lung, the mediastinum, and the bones one of the key metastatic sites of (non-small cell lung cancer) NSCLC [4–7]. A significant percentage of NSCLC patients will eventually develop brain metastases (BMs). Among newly diagnosed lung cancer patients approximately 10,8% present synchronous BMs [8]. According to a recent analysis of the Metropolitan Detroit Surveillance, Epidemiology and End Results (SEER) registry, the incidence of BMs in nonmetastatic NSCLC is 9% [9] and there is an increased incidence with more advanced stages of disease [10]. Moreover, the majority of BMs of unknown origin are eventually found to have a lung primary lesion [11, 12]. One out of four patients with anaplastic lymphoma kinase- (ALK-) rearrangement and epidermal growth factor receptor (EGFR) mutation diagnosed at an advanced stage present with BMs and prevalence increases with time [13, 14]. Patients with ALK-rearranged and EGFR-mutated NSCLC present with delayed onset of BM and have a prolonged survival compared to patients lacking these genetic alterations [15].

The median survival of patients with BMs has improved during the last two decades. According to an update of the graded prognostic assessment (GPA) for lung cancer using molecular markers (Lung-molGPA) the median survival of patients with BMs based on a database of patients diagnosed between 2006 and 2014 ranges from approximately 3 to 46.8 months depending on clinical, histological, and molecular prognostic factors. The median survival rates for adenocarcinoma and nonadenocarcinoma lung cancer are 15.2 and 9.2 months, respectively [16]. For the previous GPA, based on a population diagnosed between 1985 and 2005, median survival ranged from 3.0 to 14.8 months [17]. In the population of patients diagnosed between 1979 and 1993 which formed the database for the recursive partitioning analysis (RPA) in the seminal paper of Gaspar et al. the median survival ranged from 2 to 7 months [18]. Even though, traditionally, BMs are considered to have a very poor survival, survival analyses by metastatic site show that BMs do not carry as poor a prognosis as liver, adrenal, or even bone metastases [6, 7] and survival is primarily dependent on the number and not the location of metastatic sites [19]. The 5-year survival rate in patients with BM from NSCLC is estimated around 2.9%, which is higher than that of melanoma and renal cell cancer, approximately 2.3%, and breast cancer, with a 5-year survival rate of only 1.3% [20].

Immunotherapy has been very fruitful for NSCLC patients. Programmed death receptor-1 (PD-1) and programmed death receptor ligand-1 (PD-L1) inhibitors are considered the standard of care, especially for those patients who do not harbor a mutation targetable with tyrosine-kinase inhibitors (TKIs). Immunotherapy has the advantage of procuring very lasting results for responders, but, on the other hand, roughly only a third of patients will respond. Strategies to increase the response rate are being investigated. Evidence of enhanced response with the combination of radiation therapy and immunotherapy has attracted a lot of attention and many preclinical and clinical studies are underway in an effort to establish the connection and to explore the conditions maximizing this effect. In regard to BMs, immunotherapy has shown efficacy in brain tumors, as have targeted therapies with TKIs, in selected subgroups. Their importance for the majority of patients with BMs, however, has to be put in perspective of an equally significant progress in local treatments, surgery, and radiation therapy.

## 2. Surgical Resection

It is common practice to treat solitary or single BM in patients with good performance status and controlled extracranial disease with surgery and postoperative radiation therapy, usually SRS to the resection cavity [21]. Resection also has a role in immediately alleviating symptoms caused by a tumor in an eloquent area of the brain, a tumor of important dimensions, or a large edema. Smaller tumors, with a maximum 3-4 cm of diameter, can also be treated with stereotactic radiotherapy (SRT), either at a single fraction or in multiple fractions [22–25]. Tumors in eloquent areas of the brain were previously considered difficult to treat with either surgery or SRT. Newer techniques, however, have made this possible in centers of expertise, with stereotactic fractionated radiation therapy and microsurgical techniques [26–28]. The extent of resection can contribute to further decrease of local recurrence and neurosurgical techniques. The use of the fluorescent marker 5-aminolevulinic acid, discriminating tumor infiltration from healthy brain tissue, can contribute to the oncologic outcome [29, 30].

There is no high level of evidence up to date of the superiority of combining surgery with whole-brain radiotherapy (WBRT) over WBRT alone [31]. Two randomized controlled studies comparing surgical resection of a single brain metastasis followed by WBRT to WBRT alone favor the combined approach, while a third one failed to show a difference in survival [32–34]. In the study of Patchell et al., the presence of selection bias due to the recruitment of patients referred to a neurosurgical service may have influenced the results in favor of surgery [34, 35]. However, there was no selection bias in the other two studies, and, still, their results are contradicting. The study that did not show a survival advantage for surgery had an important percentage of patients with poor PS and extracranial metastases, for whom the addition of surgery could not offer a survival advantage, as shown in the study of Vacht et al. Of note, all three studies were published more than two decades ago, when systemic treatments were used only in a small proportion of the patient population of these studies, as is documented by Patchell et al. [32]. As the number of participants was very small, the studies were underpowered. The only safe conclusion to be drawn is the importance of good PS and stable systemic disease in order to consider a patient with BMs for surgery.

Inarguably, surgery is the unique method to obtain brain tumor tissue. Not only is this the way to safely establish a diagnosis, but also it provides new possibilities in the era of molecular and personalized oncology. The studies of Brastianos et al. and Paik et al. demonstrate the genetic heterogeneity between primary tumors and brain metastases and that genetic alteration specific to the metastatic site is of potential clinical significance [36, 37]. Thus, tumor tissue from the brain lesion can guide the choice of systemic treatment based on BM-specific genetic alterations. Cerebrospinal fluid samples could be an alternative method to detect clinically significant genetic alterations, but this has also to be validated by brain tissue samples [38, 39].

## 3. Radiation Therapy

Radiation therapy has traditionally been considered solely a local antineoplastic treatment. The main mechanism of action of radiotherapy has long thought to be the induction of DNA damage, triggering DNA damage-response pathways leading to tumor cell apoptosis, mitotic catastrophe, and senescence, as reviewed by Khanna et al. and Eriksson et al. [40, 41]. Accumulating evidence of an immunomodulatory effect of radiotherapy supports its systemic role in cancer therapy. The idea that the immune system has a central role in the tumor response to radiotherapy dates back to 1979, when Slone et al. demonstrated a differential response to radiation depending on the immunosuppression or immune stimulation of the host [42]. The first of a number of case reports of abscopal phenomena, i.e., regression of neoplastic lesions at a distance from the irradiated volume, was documented by Mole et al. [43]. With the advent of immunotherapies, there has been renewed interest in the effect of radiotherapy on the tumor microenvironment and, especially, on the immune system. Tumor cell death by high dose irradiation in SRT cannot be explained only by the direct cell death caused by DNA double-strand break and although the linear-quadratic model is applicable, it is not sufficient on its own to describe the immunogenic cell death and the cell death that results from vascular destruction, as observed after large-dose fractions [44–46]. The original work of Diamant et al. shows that the dose in an area 3 cm thick outside the PTV for stage I NSCLC patients treated with SRT is correlated with the rate of distant metastasis but not the rate of local control, suggesting a dose-dependent immunogenic effect of radiation to the tumor's microenvironment [47].

WBRT was given in the past to the majority of BM patients with an intent to offer palliation and prolong survival by a few months [48]. A 1-month median survival in untreated BM was initially improved with the use of corticosteroids by one month and WBRT managed to extend the median survival to 3-6 months [49, 50]. Dose finding trials failed to improve survival with increased dose or altered fractionation schedules compared to the standard fractionation of 30 Gy in 10 fractions [51–54]. A big retrospective study comparing different fractionations used in different countries between 1992 and 2005 found the standard fractionation to be equivalent in terms of survival with a shorter schedule of 20 Gy in 5 fractions over 1 week [55]. Ultrashort fractionation schedules of one fraction of 10 Gy or two fractions of 6 Gy have inferior outcomes, especially in regard to the duration of palliation and neurological improvement and in patients with good prognosis [56]. It should be underlined, however, that the above studies have not examined the effect of fractionation on long-term neurotoxicity.

Nowadays, WBRT is giving way to SRT exactly on the basis of improved cognitive function. SRT is the new standard of care for patients with good PS and up to 10 brain lesions with a diameter smaller than 3 cm for the largest lesion [57]. Postoperatively, SRT has replaced WBRT on the basis of a better long-term toxicity profile and an equivalent OS, in spite of an inferior local and regional (distant intracranial) control. SRT has the advantage of a minimal neurocognitive dysfunction compared to WBRT [58–61]. Salvage SRT is another treatment option that has been proposed as noninferior to adjuvant WBRT [62].

At the other end of the spectrum lie patients with a very poor prognosis, with an expected survival from diagnosis of less than 3 months, for which the QUARTZ trial showed equivalent survival and quality of life with optimum supportive care compared to WBRT 20 Gy in 5 fractions. As a result, OSC can replace WBRT in NSCLC patients with RPA class II and III with extracranial metastases or active lung disease that has failed to be controlled with systemic treatments whose BM is inoperable and SRS/SFRT is inappropriate. It should be, however, noted that one-third and one-fifth of patients in the OSC only and in the WBRT plus OSC arm, respectively, received additional anticancer therapy, mainly thoracic radiotherapy [63]. WBRT still has a place as a treatment for patients that are not candidates for either surgery or SRS/SBRT but for whom a benefit from WBRT can be anticipated, young patients with good KPS, or patients whose systemic disease is well controlled or for whom effective systemic options still exist.

WBRT also has a role for selected patients as an adjuvant treatment after either surgery or stereotactic radiotherapy. Even if the preferred treatment after surgery is SRT to the resection cavity, WBRT can also be considered, for example, in cases where the target volume would pose an increased toxicity risk. Adjuvant WBRT after SRT has been largely abandoned for patients with up to 4 BM, as a number of randomized controlled trials and a meta-analysis concluded that SRT alone could provide superior quality of life with less memory loss and less neurological dysfunction without inferior OS or functional independence, albeit at an increased risk of intracranial failure [64–68]. Recently, however, a secondary analysis of the JROSG 99-1 trial provides evidence in favor of WBRT in NSCLC patients of good prognosis. Adjuvant WBRT significantly improved OS for patients with a NSCLC primary and DS-GPA score of 2.5 to 4.0 [69]. Combined treatment provides a survival benefit over WBRT as well, in patients with a GPA of 3.5-4.0, reinforcing the previous results [70, 71].

## 4. Classical Chemotherapeutic Agents

Systemic treatments have increasingly been used in the setting of BM. Classical chemotherapy drugs, even those penetrating the BBB, like temozolomide, lack clinically significant activity for patients with BMs. Studies of WBRT in combination with chemotherapeutic agents have failed to show efficacy, possibly due to poor blood-brain barrier (BBB) penetration [72]. Pemetrexed has, however, shown some activity [73–75].

## 5. Antiangiogenic Agents

Bevacizumab (BEV) is a well-established anti-VEGF treatment for advanced and metastatic nonsquamous NSCLC. However, the trial that established the addition of BEV to pemetrexed- carboplatin and its use as maintenance treatment excluded patients with BMs [76]. A retrospective analysis of another trial that had showed only progression-free survival improvement with the addition of BEV to gemcitabine-cisplatin chemotherapy in the same patient population [77] found a statistically significant reduction in BMs in the BEV arm. Ilhan-Mutlu et al. analyzed two randomized controlled trials on breast cancer but failed to show a preventive role of BEV on BM formation. The same preventive role of BEV on intracranial metastases only was shown in an animal model of NSCLC [78]. Real time* in vivo *imaging of brain metastasis formation in a mouse model confirms that VEGF-A inhibition induced dormancy of micrometastases from lung cancer cells but not from melanoma cells [79].

The BRAIN trial, a nonrandomized phase II study, demonstrated safety and efficacy of the first- line treatment with BEV and paclitaxel on nonsquamous NSCLC patients with untreated, asymptomatic BMs, with only one grade 1 intracranial hemorrhage, a median OS of 16.0 months, and a 6-month PFS rate of 56.5% [80]. BEV may also have a corticosteroid-sparing effect, but up to now this has been only observed in primary brain tumor trials [81]. Animal models' studies and case reports indicate that BEV has a potential role in mitigating and treating radiation necrosis [82–84].

Ramucirumab, a VEGFR-2 monoclonal antibody, is FDA approved for the second-line treatment of metastatic NSCLC in combination with docetaxel on the findings of REVEL trial that did not exclude patients with stable previously treated CNS metastases, but no data has been published on this subgroup [85]. The safety and efficacy of second-line docetaxel plus ramucirumab for NSCLC patients with asymptomatic CNS involvement will be specifically addressed in phase II RAMNITA study. However, patients previously treated with surgery or WBRT will not be eligible to participate in the trial [86].

## 6. Tyrosine-Kinase Inhibitors

EGFR TKIs are standard treatment for EGFR-mutated patients with advanced and metastatic NSCLC. They penetrate the BBB and show some CNS efficacy [87]. The third-generation oral, irreversible EFGR TKI osimertinib has been FDA approved as a first-line treatment of EGFR- mutated advanced or metastatic NSCLC with exon 19 deletions or exon 21 L858R mutations. The FLAURA phase III study estimated 18.9 months PFS in the osimertinib arm compared to 10.2 months PFS in patients receiving either gefitinib or erlotinib [HR, 0.46; 95% CI 0.37 to 0.57; P<0.001]. The patient population of this study also included asymptomatic or stable, off steroids BMs patients. According to a preplanned analysis in this subgroup, CNS objective response rates were 91% and 66% with osimertinib and 68% and 43% with other EGFR TKIs in patients with ≥ one measurable CNS metastasis and in patients with measurable and/or nonmeasurable CNS lesions, respectively. Median CNS PFS was not reached in the investigatory arm and 13.9 months in the standard arm (HR, 0.48; 95% CI, 0.26 to 0.86; P = .014) [88, 89]. In the AURA 3 trial, osimertinib was more effective than the doublet pemetrexed-platinum in second-line treatment for EGFR T790M positive patients progressing on another EGFR TKI, including patients with CNS stable disease [90, 91].

Afatinib, an oral second-generation TKI approved as first-line treatment in EGFR mutant advanced or metastatic NSCLC, improved PFS over chemotherapy doublet [92]. The prespecified subgroup analyses of LUX-Lung 3 and LUX-Lung 6 demonstrated a trend towards a PFS benefit with afatinib for asymptomatic BM patients, yet, not statistically significant (LUX-Lung 3: 11.1 versus 5.4 months, hazard ratio [HR] = 0.54, p = 0.1378; LUX-Lung 6: 8.2 versus 4.7 months, HR = 0.47, p = 0.1060). After the combined analysis of the two studies, in order to increase the sample size of BMs patients, PFS benefit of afatinib versus chemotherapy was significant (HR = 0.50, 95% CI: 0.27–0.95, p = 0.0297). Of note, the PFS benefit was even more evident in those previously treated with WBRT and those with a Del19 mutation [93].

Icotinib, a first-generation TKI approved in China, demonstrated significant CNS activity in a phase III trial comparing monotherapy with icotinib to WBRT with concurrent or sequential chemotherapy in EGFR mutant patients with three or more brain lesions, resulting in a 44% risk reduction for an event of intracranial disease progression or death and a significant decrease in serious adverse events [94].

Meta-analyses of mainly noncomparative observational studies and one RCT have compared cranial irradiation alone, TKI treatment monotherapy, and the combination of a TKI with radiation therapy but have reached contradictory conclusions [95–97].

ALK TKIs are active in the CNS, with newer drugs proving to be even more efficient in the prevention of BMs, in a population with a high incidence of intracranial metastases [98]. Crizotinib, an oral TKI for the first-line treatment of advanced ALK-rearranged NSCLC, has been evaluated for its efficacy in asymptomatic BMs. This subgroup consisted of 31% of the combined study population. A correlation was found between intracranial and extracranial disease control at 12 weeks. In a comparison between patients with and without previous brain radiotherapy, the latter group had an improved intracranial control rate and median intracranial time to progression [99]. A median survival of 49.5 months is reached in patients with ALK rearrangement treated with brain radiotherapy and tyrosine-kinase inhibitor (TKI) therapy [100].

## 7. Immune Checkpoint Inhibitors

The concept that the brain is an immune-privileged site has been recently revisited with the demonstration of the presence of lymphatic vessels in the dura mater draining cerebrospinal fluid into extracranial deep cervical lymph nodes, changing our perception of the anatomy of the CNS [101]. However, it still stands true that, compared to peripheral tissues, there is a paucity of both innate and adaptive immune responses in the CNS [102]. However, activated circulating CD4+T cells have been shown to cross the blood-brain barrier and upon recognition of their cognate antigen on antigen presenting cells they induce local T cell activation, release of cytokines, and further recruitment of immune cells, eventually altering the BBB permeability characteristics, as reviewed by Engelhardt et al. [102]. Tumors develop mechanisms to evade the innate immune system, promoting immune tolerance, which is the exact target of immune checkpoint pathway inhibitors. PD-1 activated by its ligand, PD-L1, negatively regulates immune response. Currently, four such drugs have been approved for patients with NSCLC: the anti-PD-L1 drugs, atezolizumab and durvalumab, and the anti-PD-1 agents, nivolumab and pembrolizumab.

Atezolizumab improves PFS and OS when added to bevacizumab and chemotherapy with carboplatin and paclitaxel as a first-line treatment for metastatic nonsquamous NSCLC patients without EFGR mutations or ALK alterations [103]. Atezolizumab has FDA approval as a second-line treatment based on the results of a phase II trial, later validated by the OAK phase III trial, proving improved efficacy over treatment with docetaxel for advanced and metastatic NSCLC progressing on previous treatment [104, 105]. A subgroup analysis of 85 patients of the OAK trial with asymptomatic and stable BM found an improved OS with a median OS of 20.1 months for patients receiving atezolizumab over 11.9 months for patients receiving docetaxel (HR 0.54 [95%CI 0.63-0.89]) [106]. A pooled analysis from 4 studies with atezolizumab monotherapy as second-line treatment and beyond identified 27 patients with BMs, 4 asymptomatic and untreated and 23 stable and previously treated with radiation to the brain. Serious and any grade adverse events occurred in 38% and 96% of patients without baseline BMs and in 33% and 96% of patients with baseline BMs. The incidence of treatment-related neurological adverse events was 9% in the non-BMs cohort and 15% in patients with BMs at baseline, indicating that atezolizumab is well-tolerated in this cohort of patients [107].

Nivolumab is an approved second-line treatment for both squamous and nonsquamous NSCLC after proving to increase OS over docetaxel. In nonsquamous NSCLC, the CheckMate 057 trial demonstrated that nivolumab improved the 1-year and 18-month OS regardless of PD-L1 expression level but had improved outcomes with increased levels of tumor-membrane expression of the PD-1 ligand. Patients with active CNS disease were excluded, but patients treated with brain irradiation and in small corticosteroid doses without neurological symptoms, except for treatment-related adverse events, could be included [108]. In squamous NSCLC, the results of the CheckMate 017 trial favored nivolumab over docetaxel in terms of both OS and PFS across all PD-L1 expression level subgroups. This trial had the same exclusion criteria as CheckMate 057 concerning patients with CNS metastases [109].

A multicenter study in Japan retrospectively examined data from all patients receiving nivolumab between December 2015 and July 2016 to determine the predictive significance of metastatic site on nivolumab efficacy in a real-world environment. Of 201 patients treated, 51 (25.4%) had brain metastases. No additional data on these patients concerning extent of intracranial disease, prior radiation therapy, symptoms, or corticosteroid use are given. In spite of the limitations of this study, it is interesting to note that a quarter of patients treated had BMs. The investigators concluded that the only factors independently associated with a shorter PFS were poor ECOG PS and liver and lung metastases [110]. In a similar real-world data study, poor PS is again associated with prognosis, but, contrary to the previous study, brain was the metastatic site associated with poor prognosis with nivolumab [111]. Another retrospective multicenter study was conducted in France in order to collect data concerning intracranial activity and safety of nivolumab in NSCLC patients. The study included 43 BM patients, 37% of whom had active intracranial disease. Intracranial activity was found similar with extracranial efficacy, with an acceptable toxicity profile [112]. Further support of effectiveness in a real-world setting for second-line treatment with nivolumab comes from a 260-patient series from Israel, 21% of whom had BM. No serious neurologic adverse event was observed [113].

Pembrolizumab is approved in metastatic nonsquamous NSCLC patients without EGFR or ALK genomic tumor aberrations, irrespective of PD-L1 expression, as a first-line treatment in combination with pemetrexed and carboplatin on the basis of phase I/II KEYNOTE-021 study [114]. Pembrolizumab can also be given as monotherapy for the first-line treatment of NSCLC patients with PD-L1 expression on ≥ 50% of tumor cells as the phase III KEYNOTE-024 study showed that it improved PFS and OS and had a better toxicity profile compared to platinum-based chemotherapy [115]. The updated analysis, with a median follow-up of 24 months, demonstrated that the significant benefit with the addition of pembrolizumab was sustained with HR for OS of 0.56 (95% CI, 0.32-0.95, p=0.0151) [116]. Its effectiveness for PD-L1 positive patients on second-line treatment and beyond has been assessed in the phase II/III KEYNOTE-010 trial, which established pembrolizumab in this treatment population [117].

The role of pembrolizumab in patients without neurologic symptoms, perilesional edema, leptomeningeal disease, or the need for corticosteroids, with at least one untreated or progressive BM between 5 and 20 mm, has been addressed in phase II trial that enrolled 18 melanoma and 34 PD-L1 positive NSCLC patients without previous treatment with anti-PD-1/PD-L1 agents. An intracranial response was achieved in 22% of melanoma patients and 33% of NSCLC patients, and most responses were durable. The authors conclude that pembrolizumab was well-tolerated and showed promising efficacy, with high concordance between intracranial and extracranial responses [118]. A retrospective cohort study from a tertiary oncological center published its results from the combination of carboplatin and pemetrexed with (cohort A) or without (cohort B) pembrolizumab, indicating a potential benefit with the addition of pembrolizumab, which also applied to patients with BMs [119].

## 8. Synergy between Radiation Therapy and Immune Checkpoint Inhibitors

An increasing number of case reports describe an abscopal effect of radiation therapy combined or not to immunotherapy [120–122]. Although in the reports of combined treatments one cannot rule out the possibility of a delayed response to immunotherapy, the existence of an off-target effect with radiotherapy alone acts as a proof of principle of the immunogenic role of radiation. In NSLC, reports of the effect are scarce [123–128]; however, there is strong evidence of a synergy between radiation therapy and immunotherapy. Preclinical evidence of an increase in tumor PD-L1 expression by radiation therapy, as reviewed in reference [129], has been recently confirmed clinically in 46 stage II and III soft tissue sarcomas patients treated with preoperative RT. PD-L1 expression was measured before and after radiation and was found increased (> 1%) in 10.9% of patients after RT compared to no patient with an increased PD-L1 before RT [130].

A secondary analysis of a phase I trial of pembrolizumab in advanced NSCLC patients (KEYNOTE-001) demonstrated a statistically significant increase in the PFS and OS of patients pretreated with radiation therapy (HR 0.56 [95%CI 0.34-0.91], p=0.019 and HR 0.58 [95%CI 0.36-0.94], p=0.026, respectively). Previous radiotherapy and previous extracranial radiotherapy were the only independent predictors of prolonging overall survival. A separate analysis of pulmonary toxicities between the two groups found no difference in serious pulmonary adverse events between patients with or without previous thoracic irradiation [131].

Durvalumab, an antiprogrammed death ligand 1 antibody, has recently been approved as consolidation therapy in unresectable stage III NSCLC patients previously treated with concurrent chemoradiation on the basis of the PACIFIC trial that demonstrated that durvalumab treated patients had an improved PFS compared to those treated with placebo [132]. Taking into consideration the conclusions of the secondary analysis of KEYNOTE-001, it is possible that the synergy between durvalumab and previous RT contributed to the results of the PACIFIC trial. A major concern in the combination of RT with immune checkpoint inhibitors is the increase in pneumonitis, but the toxicity of durvalumab after radiation was deemed acceptable. This can be partly explained by the results of two meta-analyses that have shown a decreased incidence of immune-related adverse events and pneumonitis with PD-L1 inhibitors compared to PD-1 antibodies [133, 134].

A retrospective study reported on 260 patients with NSCLC, melanoma, and renal cell carcinoma who were treated for BMs with SRT, without prior WBRT, and immune checkpoint inhibitors, ipilimumab, nivolumab, or pembrolizumab. Concurrent use of ICI was defined as given within two weeks of SRR/SRT. Median OS was 12.9 months, 14.5 months, and 24.7 months for patients treated with SRS/SRT alone, nonconcurrent SRT, and immune checkpoint inhibitors and concurrent treatment, respectively. On multivariate analysis, concurrent use of immune checkpoint inhibitors was associated favorably with OS compared with the other two treatment strategies, without increasing the rate of adverse events [135]. Similarly, Shapira et al. reviewed the medical records of 37 NSCLC patients treated with SRT and PD-1 pathway inhibitors for BMs between 2012 and 2017 in a single institution. Concurrent instead of sequential treatment was associated with higher rates of OS and LC and lower rates of distant brain failure at 1 year [136]. The retrospective analysis of 17 patients treated with SRS to 49 brain lesions either before, during, or after anti-PD-1/PD-L1 treatment (nivolumab or durvalumab) suggests good tolerance of the combined treatment and an improved distant brain control when radiation precedes or is given concomitantly with systemic treatment [137].

## 9. Palliative Care

As life expectancy is relatively short, quality of life and preservation of neurological function are a priority in patients with BMs. Specialized palliative care should address the needs of BMs patients, which differ from those of the general oncologic population [138]. An early integration of palliative care with the patient's oncologic treatment is key to a successful intervention and a better use of health resources [139]. There is consensus that anticonvulsants should not be prescribed prophylactically as they do not prevent the onset of seizures [140]. Corticosteroids, most commonly dexamethasone, are used in symptomatic patients to reduce cerebral edema and improve neurologic deficits. Doses should be kept as low as possible and protracted tapering should be avoided, as side-effects such as sleep disturbances, mood disorders, myopathy, osteoporosis, and weight gain are dose-dependent [141]. Recently, there has been accumulating evidence that daily doses higher than the equivalent of 10 mg of prednisone can limit the efficacy of immunotherapy, further stressing the need to refrain from high corticosteroid doses [142, 143]. Up to date, alternative agents to alleviate cerebral edema do not have an established role to substitute corticosteroids in clinical practice. Bevacizumab has a clear antiedema effect and can be used in patients suffering from serious steroids side-effects or refractory to corticosteroids [141].

## 10. Conclusion

BMs have been treated relatively homogenously for decades, with WBRT being the standard of care. Only a small percentage of patients, those with limited intracranial disease and RPA class I, could benefit from the addition of surgery or SRS. In the last few years, many advances in both systemic and local therapies for the treatment of advanced and metastatic non-small cell lung cancer have come to light. Surgical and radiation techniques become more elaborate and accurate, enabling greater sparing of healthy tissue surrounding the tumor, preserving neurological function, and, at the same time, achieving greater elimination of macroscopic disease. The ways in which they can be better combined are still a matter of debate. Particularly, neurocognitive dysfunction, caused by WBRT, has become increasingly important, ahead of that of intracranial control, as patients live longer with targeted agents and immunomodulatory drugs. However, WBRT still has the advantage of a better local control, which may be translated also in a survival advantage in the future, if distant disease will be managed more effectively by systemic treatments.

Neuroprotective agents have been vigorously investigated in the hope that WBRT could be administered without affecting memory and learning processes. Memantine, tested in a phase III trial, is such a neuroprotective agent, but it failed to produce a statistically significant amelioration in cognitive function, possibly due to patient loss. However, there was a clear improvement in some aspects, as in processing speed, executive function, and delayed recognition [144]. With the same intent of preserving cognition, WBRT has used intensity-modulated radiotherapy (IMRT), to avoid the hippocampus, a technique currently evaluated in a prospective clinical trial (NCT02147028). Furthermore, the fractionation studies of the past had not been done with intent of sparing memory loss, and there is a possible interest in reexamining the effect of fractionation in the modern population of BM patients, with many more asymptomatic patients as a result of frequent MRI scans, and the concurrent use of systemic agents. Of note, the QUARTZ trial, by using a hypofractionated regimen of 20 Gy in 5 fractions, cannot conclude that its results would be the same if the fractionation of 30 Gy in 10 fractions had been used.

One of the greatest breakthroughs in modern oncology is the realization of how radiation therapy not only acts as an ablative local mechanism on tumor cells and its vasculature, but also has a systemic effect through the induction of an immunogenic cell death. The increasing awareness of the effect radiation therapy can have at a distance from the irradiated volume can lead to the exploitation of old tools in new ways. Immunotherapy trials in lung cancer are performing retrospective or preplanned analyses of treatment response according to previous radiation treatment and show convincing evidence in the synergy between radiotherapy and immunotherapy in a clinical setting. Further research is necessary in the dosing, sequencing, and timing of treatments in order to maximize the benefit.

Patients with CNS metastases have largely been excluded from the clinical trials that have changed the landscape in NSCLC therapy. At present this has started to change, but, still, only BM patients with controlled or asymptomatic intracranial disease are included. A combination of treatments, including surgery, stereotactic radiotherapy, WBRT, and systemic therapies, can be used for intracranial metastases with the intent of palliation of symptoms, preservation of neurocognitive function and quality of life, and possibly prolongation of survival. Only multidisciplinary designed clinical trials can address the clinical challenge posed by BMs. The optimum treatment management of these patients can only be decided in a multidisciplinary team. The extent of extracranial disease should be weighed against the risk of intracranial progression to inform a collective decision on the choice and sequencing of treatments.
